# The use of cangrelor with heparin for left ventricular assist device implantation in a patient with acute heparin-induced thrombocytopenia

**DOI:** 10.1186/s13019-018-0721-x

**Published:** 2018-04-17

**Authors:** Yan K. Gernhofer, Michael Ross, Swapnil Khoche, Victor Pretorius

**Affiliations:** 1 Department of Surgery, Division of Cardiothoracic Surgery, University of California San Diego, Sulpizio Cardiovascular Center, 9434 Medical Center Drive, La Jolla, CA 92093 USA; 2 Department of Anesthesiology, University of California San Diego, Sulpizio Cardiovascular Center, La Jolla, CA USA

**Keywords:** Left ventricular assist device, LVAD, Heparin-induced thrombocytopenia, HIT, Cardiopulmonary bypass, CPB, Cangrelor, Antiplatelet agent

## Abstract

**Background:**

Optimal anticoagulation strategy for cardiopulmonary bypass (CPB) in end-stage heart failure patients with heparin-induced thrombocytopenia (HIT) requiring left ventricular assist device (LVAD) implantation remains uncertain. Presently, there are no large-scale randomized studies comparing outcomes of alternative anticoagulation strategies for CPB in this patient population. A novel antiplatelet agent – cangrelor, which is a potent P2Y12 inhibitor with robust antiplatelet efficacy, rapid reversibility, and measurable drug effect, has become available since 2015. Intraoperative anticoagulation for CPB using cangrelor with heparin has not been reported before.

**Case presentation:**

We report the case of a 47-year-old male with ischemic cardiomyopathy and acute HIT, who underwent an urgent LVAD implantation using cangrelor with heparin for anticoagulation on CPB. This novel strategy resulted in satisfactory anticoagulation for CPB without perioperative thromboembolic events or major bleeding requiring reoperation.

**Conclusions:**

**C**angrelor with heparin was an effective anticoagulation strategy for CPB in this critically ill patient with acute HIT requiring an urgent LVAD implantation. Further studies are warranted to evaluate its efficacy and replicability in other patients with acute or subacute HIT who require urgent cardiac surgery.

## Background

Heparin-induced thrombocytopenia (HIT) is an immune-mediated adverse drug reaction that occurs on re-exposure to unfractionated heparin and, less commonly, other heparin derivatives. It is associated with significant risk of venous and arterial thromboembolic complications and major bleeding [1, 2]. Prolonged exposure to heparin has been identified as one of the risk factors for HIT [3]. End-stage heart failure (HF) patients are frequently exposed to heparin for prevention of intravascular thrombosis during hospitalizations and diagnostic or interventional procedures. They are therefore at increased risk of developing HIT. In end-stage HF patients with acute or subacute HIT requiring urgent LVAD implantation, alternative anticoagulation strategy for cardiopulmonary bypass (CPB) must be used. This case report describes the first successful use of cangrelor with heparin for CPB in a patient with acute HIT undergoing an urgent LVAD implantation.

## Case presentation

A 47-year-old male (58 kg, 178 cm) with ischemic cardiomyopathy, left ventricular ejection fraction of 10%, congestive hepatopathy, anasarca, and cardiac cachexia was transferred to our institution for consideration of advanced therapies. Pertinent past medical history included ST-elevation myocardial infarction, post multiple percutaneous coronary interventions (last coronary stents placed approximately 5 months prior), chronic obstructive pulmonary disease, and recently diagnosed left lower extremity deep vein thrombosis (DVT). Important home medications included clopidogrel 75 mg and apixaban 5 mg daily. Clopidogrel was continued on admission, while anticoagulation for DVT was maintained with a heparin infusion instead of apixaban.

Over the next 5 days, despite optimal medical therapy, he developed cardiogenic shock requiring emergent intra-aortic balloon pump placement and subsequent percutaneous Impella CP® device placement. Initial platelet count of 87 × 10^3^/μL decreased to 32 × 10^3^/μL after 8 days of heparin infusion. His 4Ts score was intermediate, and heparin-platelet factor 4 enzyme-linked immunosorbent assay (PF4 ELISA) was strongly positive with an optical density of 3.3. Heparin was replaced with bivalirudin infusion due to high suspicion of HIT.

The serotonin release assay (SRA) test was performed with the result unavailable preoperatively. Although later reported negative, the validity of the test was questionable as it was performed while the patient was on clopidogrel with a P2Y12 platelet reactivity units (PRU) test of 104 at the time. The diagnosis of HIT in this patient was established on the basis of significant HIT antibody positivity and clinical 4Ts score.

After 3 days on bivalirudin treatment, the patient’s platelet count remained stable at 40 × 10^3^/μL. His P2Y12 PRU test result finally rose > 200 after discontinuation of clopidogrel, indicating return of normal platelet function. Thus, he was taken for urgent HeartMate 3® LVAD implantation and removal of percutaneous Impella CP® device that day. Bivalirudin infusion was stopped on call to the operating room, and cangrelor with heparin was used for anticoagulation on CPB. Following a midline sternotomy and creation of LVAD driveline tract, a loading dose of cangrelor (30 mcg/kg) was administered over a minute, followed immediately by a continuous infusion of cangrelor at 4 mcg/kg/min. First intraoperative P2Y12 PRU test was drawn prior to heparin administration. Full dose heparin (300 units/kg) was given 10 min after cangrelor administration. Once activated clotting time (ACT) reached above 450 s, full CPB was commenced after cannulation. ACT was monitored every 30 min and maintained at a goal of > 450 s throughout CPB.

After implantation of LVAD, the patient was successfully weaned from CPB. Cangrelor infusion was discontinued 10 min prior to heparin reversal with protamine. Second intraoperative P2Y12 PRU test was drawn after discontinuation of cangrelor. In addition to transfusion of 2 units of platelets to promote return of platelet function, 2 units of packed red blood cells (PRBC) and 2 units of fresh frozen plasma (FFP) were also transfused prior to chest closure. Final intraoperative P2Y12 PRU test was drawn following blood product transfusion. Postoperative thromboelastography revealed satisfactory coagulation status. The patient was then taken to the intensive care unit on intravenous dobutamine 8 mcg/kg/min, epinephrine 0.02 mcg/kg/min, vasopressin 0.04 units/min, norepinephrine 5 mcg/min, and 40 ppm of inhaled nitric oxide. The HeartMate 3® LVAD was set at 4900 rpm, yielding a flow of 3.8 L/min.

Patient was coagulopathic due to impaired liver function. Fibrinogen was 180 mg/dL and international normalized ratio (INR) was 1.7 on postoperative day 1, for which he received transfusion of 2 units PRBC, 2 units of FFP, and 10 packs of cryoprecipitate. No platelet transfusion was given as platelet count was 102 × 10^3^/μL. Anticoagulation with bivalirudin was eventually restarted on postoperative day 2. Transition to warfarin therapy was achieved by postoperative day 13, with a goal INR of 2–3. The patient’s platelet count recovered to > 150 × 10^3^/μL by postoperative day 4 and remained within normal limits thereafter. There were no thromboembolic complications or major bleeding during his postoperative course, and he was discharged home on postoperative day 35. He continues to do well on device support.

## Discussion and conclusions

HIT results from the development of IgG antibodies directed to the complex of heparin-PF4. They form multi-molecular IgG-heparin-PF4 complexes, which bind to FcγRII receptors on platelet surface and crosslink them. This induces intense platelet activation, massive thrombin generation, and release of highly coagulable micro particles that promote thrombosis [4]. There are multiple phases of HIT: suspected, acute, subacute, and remote [5]. Patients with acute HIT are at markedly increased risk of thromboembolic complications. Mortality rates even with treatment have been reported as high as 25% [6]. Management of patients with acute HIT requiring urgent cardiac surgery on CPB presents a serious challenge.

According to the current 9th American College of Chest Physician (ACCP) Guidelines, the use of bivalirudin, a highly specific inhibitor of circulating or clot bound thrombin, is recommended over other nonheparin anticoagulants or heparin plus antiplatelet agents in patients with acute HIT undergoing urgent cardiac surgery (Grade 2C) [3]. However, there are shortcomings with using bivalirudin for CPB. First, there is no specific test to monitor for level of anticoagulation, making maintenance of therapeutic anticoagulation unpredictable. Second, there is no known reversal agent for bivalirudin. Thirdly, bivalirudin loses efficacy in stagnant blood due to proteolytic cleavage of the drug. Modifications to bypass circuit to avoid blood stagnation and thrombus formation within the circuit are crucial. During implantation, stasis of blood inside the LVAD pump can lead to thrombus formation on the rotor or housing which might negatively affect device operation [7]. Lastly, clearance of bivalirudin is dependent on renal function. Patients with impaired renal function are at risk of bleeding complications due to significant reduction in plasma clearance of up to 24% [8]. There are case reports describing prolonged coagulopathy post LVAD implantation in HIT patients using bivalirudin [7, 9, 10].

The use of heparin has become an integral part of cardiac surgery, owing to its predictability, rapid action, reliable reversal with protamine, and accurate monitoring using ACT. An alternative anticoagulation strategy for CPB in HIT-positive patients involves the use of antiplatelet agent with heparin. Lee et al. recently reported the successful use of abciximab/heparin protocol in six HIT patients who underwent LVAD implantation [11]. The main concern with glycoprotein IIb/IIIa inhibitors such as abciximab is the increased risk of bleeding complications when compared with novel antiplatelet agent – cangrelor [12]. The antiplatelet effect of abciximab lasts up to 36 h after discontinuation. Furthermore, there is no point-of-care measurement to monitor the effect of abciximab. Without a way to monitor drug effect, it is challenging to determine adequacy of platelet inhibition, recovery of platelet function after drug discontinuation, and transfusion requirement to restore platelet function.

**Fig. 1:**
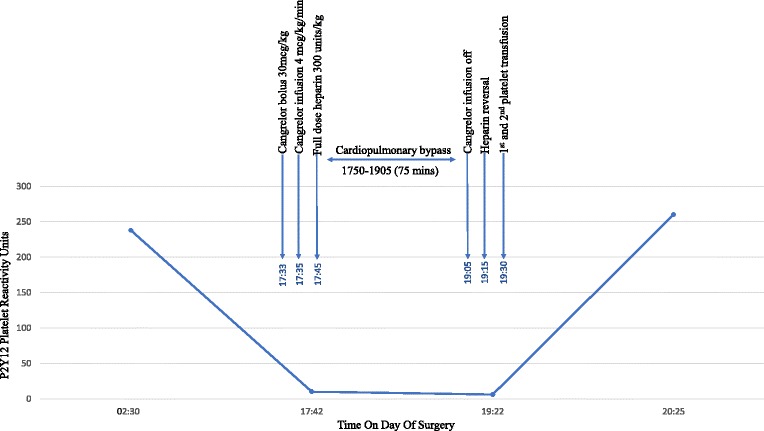
Graph of P2Y12 platelet reactivity units throughout the operation

Cangrelor is a potent, rapid-acting, reversible antagonist of the P2Y12 receptor approved by the United States Food and Drug Administration in 2015 as an intravenous anticoagulant [13]. Therapeutic dosage of cangrelor achieves platelet inhibition up to 95% within 2 min of its administration. The plasma half-life of cangrelor is approximately 3–6 min. Complete recovery of platelet function is expected within 2 h following discontinuation of infusion. Its elimination rate is not influenced by sex, age, and renal or liver function, which makes this agent ideal for patients with multiple comorbidities similar to our patient. In addition to these favorable characteristics, its drug effect can be monitored with a convenient, quick and reliable P2Y12 PRU test, allowing clinicians to make timely decisions perioperatively (Fig. 1).

This case report illustrates the first successful use of cangrelor with heparin in a patient with acute HIT undergoing an urgent LVAD implantation on CPB. Our strategy resulted in satisfactory anticoagulation for CPB without perioperative thromboembolic events or major bleeding requiring reoperation. This novel anticoagulation strategy provided us a valuable option for intraoperative anticoagulation in this critically ill patient. Further studies are warranted to evaluate its efficacy and replicability in other patients with acute or subacute HIT who require urgent cardiac surgery.

## Abbreviations

ACT: Activated clotting time
CPB: Cardiopulmonary bypass
DVT: Deep vein thrombosis
FFP: Fresh frozen plasma
HF: Heart failure
HIT: Heparin-induced thrombocytopenia
INR: International normalized ratio
PF4 ELISA: Platelet factor 4 enzyme-linked immunosorbent assay
PRBC: Paced red blood cells
PRU: Platelet reactivity units
SRA: Serotonin release assay
